# Evaluation of vascular aging on measures of cardiac function and mechanical efficiency: insights from *in-silico* modeling

**DOI:** 10.3389/fcvm.2024.1351484

**Published:** 2024-03-27

**Authors:** Lawrence J. Mulligan, Julian Thrash, Ludmil Mitrev, Douglas Folk, Alyssa Exarchakis, Daniel Ewert, Jeffrey C. Hill

**Affiliations:** ^1^ Department of Anesthesiology, Cooper University Hospital, Camden, NJ, United States; ^2^ Cooper Medical School of Rowan University, Camden, NJ, United States; ^3^ Department of Electrical and Computer Engineering, North Dakota State University, Fargo, ND, United States; ^4^ Department of Integrated Engineering, Minnesota State University Mankato, MN, United States; ^5^ Department of Biomedical Engineering, University of North Dakota, Grand Forks, ND, United States; ^6^ Department of Diagnostic Medical Sonography, School of Medical Imaging and Therapeutics, Massachusetts College of Pharmacy and Health Sciences University, Worcester, MA, United States

**Keywords:** computational model, vascular aging, mechanical efficiency, pressure-volume area, aortic compliance, ventricular-vascular coupling, stroke work, end-systolic pressure-volume relationship

## Abstract

**Introduction:**

This study evaluated the hypothesis that vascular aging (VA) reduces ventricular contractile function and mechanical efficiency (ME) using the left ventricular pressure-volume (PV) construct.

**Methods:**

A previously published *in-silico* computational model (CM) was modified to evaluate the hypothesis in two phases. In phase I, the CM included five settings of aortic compliance (*C_A_*) from normal to stiff, studied at a heart rate of 80 bpm, and phase II included the normal to stiff *C_A_* settings evaluated at 60, 100, and 140 bpm. The PV construct provided steady-state and transient data through a simulated vena caval occlusion (VCO). The steady-state data included left ventricular volumes (EDV and ESV), stroke work (SW), and VCO provided the PV area (PVA) data in addition to the three measures of contractile state (CS): end-systolic pressure-volume relationship (ESPVR), *dP/dt*_max_-EDV and preload recruitable stroke work (PRSW). Finally, ME was calculated with the SW/PVA parameter.

**Results:**

In phase I, EDV and ESV increased, as did SW and PVA. The impact on the CS parameters demonstrated a small decrease in ESPVR, no change in *dP/dt*_max_-EDV, and a large increase in PRSW. ME decreased from 71.5 to 60.8%, respectively. In phase II, at the normal and stiff *C_A_* settings, across the heart rates studied, EDV and ESV decreased, ESPVR and *dP/dt*_max_-EDV increased and PRSW decreased. ME decreased from 76.4 to 62.6% at the normal *C_A_* and 65.8 to 53.2% at the stiff *C_A_*.

**Discussion:**

The CM generated new insights regarding how the VA process impacts the contractile state of the myocardium and ME.

## 1 Introduction

Over the past five decades, methods for assessing cardiac function and aortic compliance (*C_A_*) in pre-clinical and clinical environments have enhanced our understanding of ventricular-vascular coupling (VVC) (1, 2). Several researchers have revisited the importance of *C_A_* on cardiovascular health demonstrating that a loss in *C_A_* was associated with decreases in cardiac function, increases in cardiac work and an increased transfer of arterial pulsatility (3, 7). While precise evaluation of cardiac function has primarily relied on the invasive left ventricular (LV) pressure-volume (PV) construct, noninvasive tools to evaluate *C_A_* have relied on the use of the three-element Windkessel, pulse-wave velocity (PWV), and effective arterial elastance (Ea) models (2, 8, 9). Novel MRI tools have led to important new findings regarding how age impacts the morphology and *C_A_* of the aorta (10, 11). However, like invasive measures, MRI is not an option for routine clinical use; therefore, the quest for a reliable noninvasive measure of VA has yet to be identified.

The relationship between a loss in *C_A_* during the aging process and an increase in aortic diameter, along with increases in PWV, augmentation index, and pulse pressure (PP) has gained attention, as summarized by the Framingham group (12, 13). Their results demonstrated that even in the presence of normal blood pressure, during a life span from the second to the seventh decade of life, a significant increase in PWV is observed. This pathognomonic finding is associated with an increased aortic diameter, loss in arterial distensibility, and increased wall stress, and several clinical studies assessing *C_A_* and PWV have provided evidence that supports this relationship (3, 12, 13).

The work from Kelly et al. (14) demonstrated that a decrease in *C_A_* led to changes in cardiac function and myocardial efficiency. As the study transitioned from the normal to the stiff aorta, an increase in stroke work (SW) and PP was observed, along with an increase in myocardial oxygen consumption (MVO_2_). However, they did not observe a change in the end-systolic pressure-volume relationship (ESPVR) or preload recruitable stroke work (PRSW), a load-insensitive measure of LV contractility that provides insights into the relationship between SW and end-diastolic volume (EDV). Other studies employing a range of *in-vitro* or *in-vivo* models provided the foundation for later investigations (8, 9, 15, 16). In an attempt to utilize the data collected from the LV PV construct, using Ea became a popular parameter to quantify changes in the VVC. While this parameter is derived from the first beat during preload manipulation to create ESPVR, Ea does not remain constant during the collection of LV PV data during a VCO and is a poor measure of CA (17). Work by Freeman (18), using a canine model, demonstrated that a loss in CA, with an inflation of an intraarterial balloon-tipped catheter, did not impact ESPVR or PRSW but did result in an increase in the maximal rate of rise in LV pressure (*dP/dt*_max_). At the same time, mechanical efficiency (ME) decreased.

A lesser-known variable, the PV area-end diastolic volume (PVA-EDV) relationship, increased from a normal state of VVC to one that mimics a stiff aorta. Building on these results (14) and the findings from the Framingham studies (12, 13), we modified a previously validated, lumped parameter, closed-loop, cardiovascular computational model (CM) to generate the left ventricular PV variables (19, 20). The novel CM provided associated parameters that allowed changes in *C_A_* across the normal-to-stiff spectrum of the aging aorta and provided insight into the linear nature of the CM and the impact of losses in *C_A_* on cardiac function. In addition, we used the CM to evaluate how increases in heart rate (HR) impact the variables of cardiac function.

## 2 Methods

### 2.1 Computational model

A cardiopulmonary CM was reconstructed based on a previously validated comprehensive model of the system, using the MathWorks R2020b release of MATLAB and Simulink (1 Apple Hill Drive, Natick, MA, USA) for the purpose of simulating human cardiovascular experiments (19, 20). The original lumped parameter CM includes a five-compartment systemic vascular system, pulmonary circulation, lung mechanics, and left and right ventricles and atria. The model is hemodynamically regulated through an autonomic neurological control system and local-effect autoregulation through blood-gas concentration (19, 20). Each system consists of multiple interlinked mathematical equations recreated within the Simulink environment for use as an experimental tool.

#### 2.1.1 Baseline model verification

**Figure 1:**
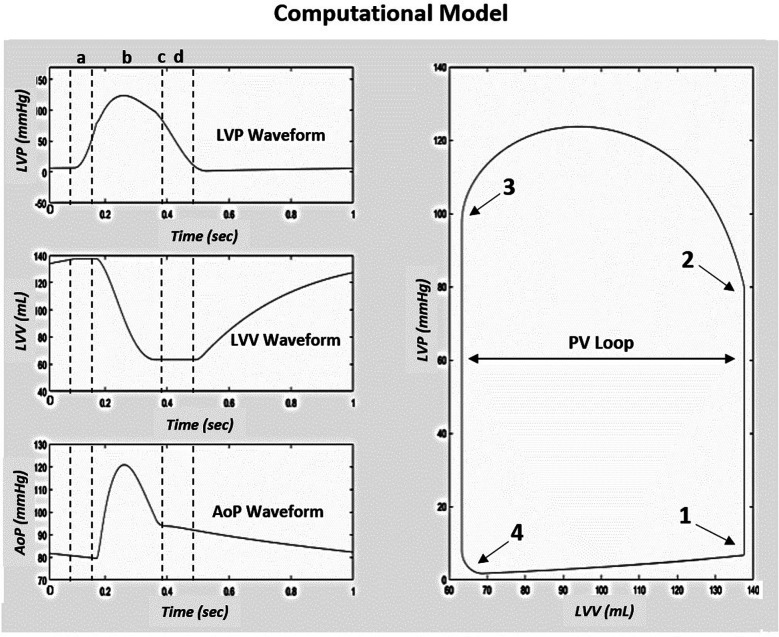
Simulink computational model native condition cardiac response modified to reproduce the original model results from (20). (a) is the IVCT, (b) is the LV ejection time, (c) is AVC, and (d) is the IVRT. To the right of the three waveforms is the corresponding PV loop generated from the model based on the cardiac event timing, (1) MVC, (2) AVO, (3) AVC, and (4) MVO. Abbreviations: LVP, left ventricular pressure; LVV, left ventricular volume; AoP, aortic pressure; PV, pressure-volume; IVCT, isovolumic contraction time; AVC, aortic valve closure; IVRT, isovolumic relaxation time; MVC, mitral valve closure; AVO, aortic valve opening; MVO, mitral valve opening.

Prior to the study, the Simulink CM integrity was assessed through a process of tuning model parameters to recreate the published results of the original CM (19, 20). Model-generated cardiovascular waveforms from the Simulink model were directly compared to the left ventricular pressure (LVP), volume (LVV), aortic pressure (AoP), and PV Loop cardiac response of the original CM (Figure 1). The hemodynamic equations and parameters of the Simulink CM were tuned to achieve a comparable response to the original CM waveforms and standard human physiologic cardiac PV responses seen in the work of C. J. Wiggers (19, 20, 21). Under normal compliance and resistance conditions, the model generated PV waveforms with systolic, diastolic, and PP of approximately 120 mm Hg, 80 mm Hg, and 40 mm Hg, respectively.

#### 2.1.2 Simulink CM arterial vascular system overview

**Figure 2:**
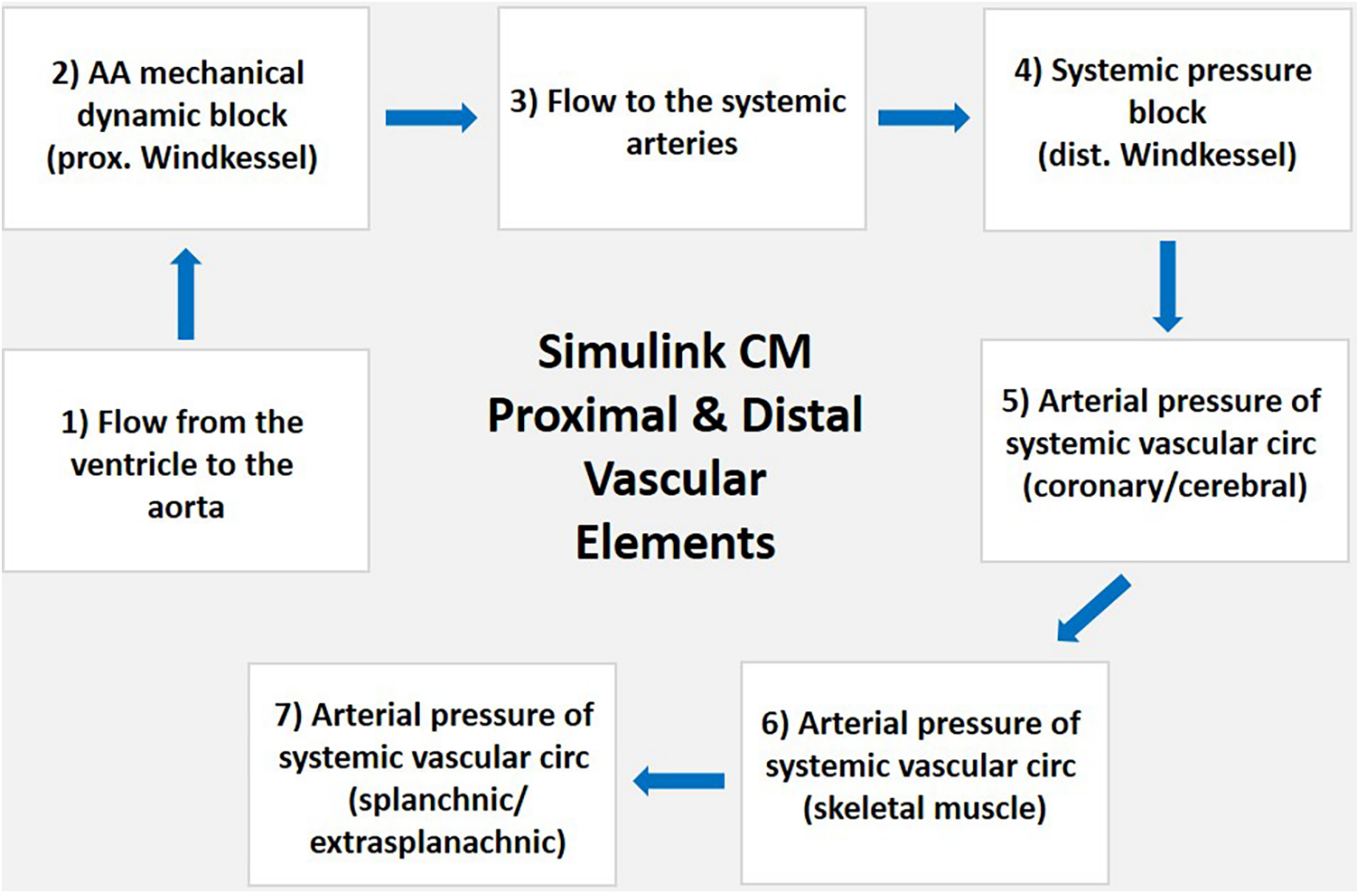
Block diagram of Simulink CM proximal and distal vascular elements. (1) Blood flows from the ventricle to the (2) AA connecting with the five compartments of the systemic vascular system starting at the parallel equivalent arterial pressure block that is the location of the proximal Windkessel model; (3) flow continues through the systemic arteries to the; (4) systemic arterial pressure block location of the distal Windkessel model; the distal element of the Windkessel model consists of a mass-flow balance differential equation that determines the arterial pressure of the five parallel regions including the; (5) coronary and cerebral; (6) skeletal muscle); and (7) splanchnic and extra-splanachnic. Each point reflects the summation of arterial blood flow through each region, and its total equivalent arterial compliance. The distal arterial regional load varies based on the autonomic and local effect-regulated hemodynamic resistances while compliance of each compartment is held constant. The proximal and distal regions of the vascular system connect in series, dictating the systemic circulation within the Simulink CM. Abbreviations. CM, computational model; AA, aortic arch; prox, proximal;, dist, distal; circ, circulation.

The structure of the vascular system within the Simulink CM consists of proximal and distal arterial regions that form a Windkessel model. The system begins with blood flowing from the left ventricle to the aortic arch, representing the Windkessel system's proximal element. Flow continues through the systemic arteries to the arterial pressure block location of the distal Windkessel model. The distal element of the Windkessel model consists of a mass-flow balance differential equation that determines the arterial pressure of the five parallel regions, including the coronary, cerebral, skeletal muscle, splanchnic, and extrasplanachnic (Figure 2). Each point reflects the summation of arterial blood flow through each region and its total equivalent arterial compliance. The distal arterial regional load varies based on the autonomic and local effect-regulated hemodynamic resistances while compliance of each compartment is held constant. The proximal and distal regions of the vascular system connect in series, dictating the systemic circulation within the Simulink CM. Vascular load is varied by modifying the aortic arch regional compliance element.

#### 2.1.3 Experimental model setup

After verification of the model was adapted to fit the experimental setup and procedure of the canine experiment seen in Kelly et al. (14). The model was modified to create the scenario used in invasive PV loop studies: right atrial pacing, autonomic nervous system blockade, and a decrease in preload that mimics the VCO. The nominal vascular compliance and peripheral resistances were directly modified to simulate normal and stiff conditions that mimic human cardiac and arterial function and physiology (19, 20). The model developed depicts the venous blood flow to the right atrium through the superior and inferior vena cava as the total summation of flow converging through the thoracic cavity veins. Flow convergence is modeled by pooling the total venous blood flow from the five vascular compartments.

#### 2.1.4 Vena caval occlusion

Occlusion of the venous return was simulated using a time-based increase in the hemodynamic resistance of the thoracic cavity veins after allowing the system to reach a steady state three minutes before the occlusion. The occlusion occurred distal to the tricuspid valve and downstream of the five systemic vein sub-compartments. This *in silico* method is equivalent to occlusion of the human vena cava via a balloon-expandable catheter delivery system that replicates a reduced venous blood flow return.

**Figure 3:**
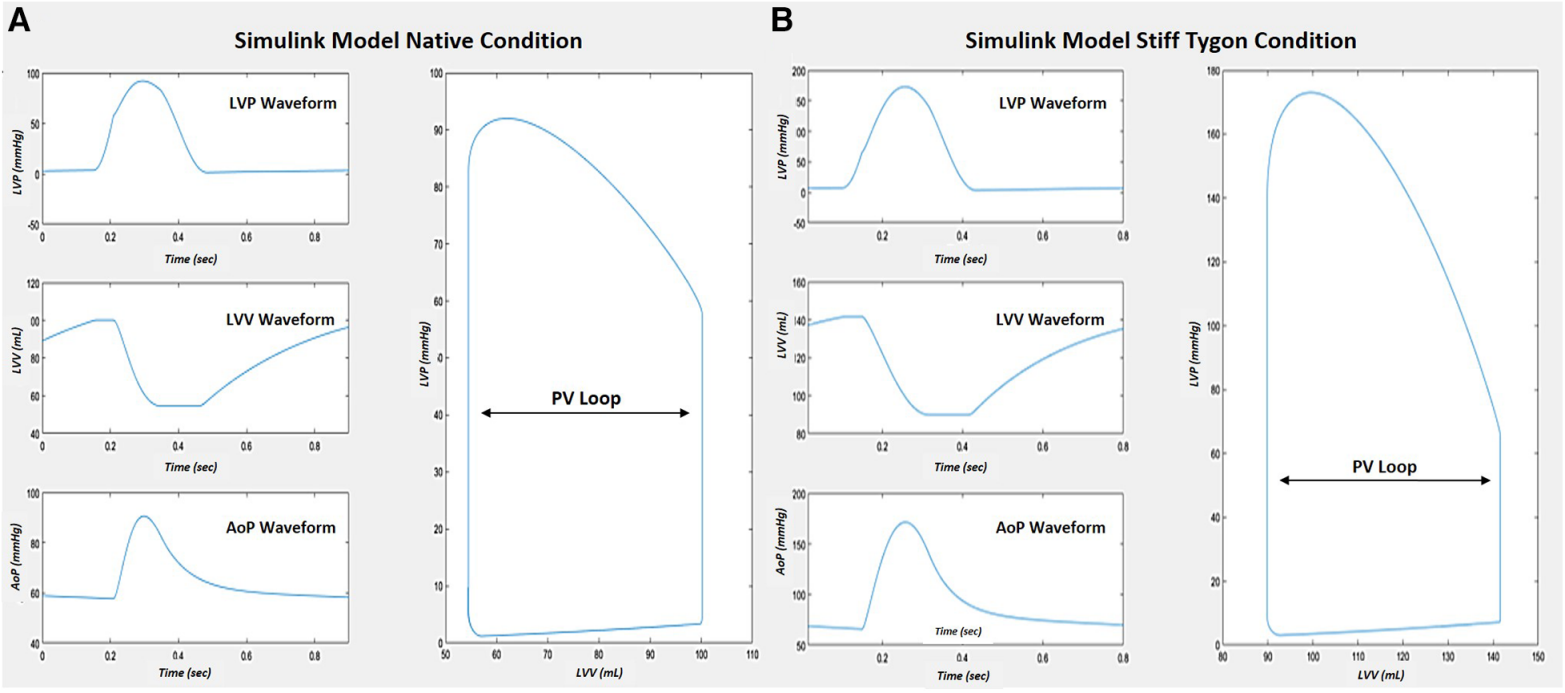
Simulink CM cardiac response comparison after tuning vascular parameters to match Kelly et al. [15] The native model demonstrated normal pressure-volume waveforms and PV loop (**A**). When the stiff Tygon condition was implemented, press-volume waveform changes resulted in a change in the PV loop (**B**).

Right atrial pacing was simulated at 80 bpm across the five levels of compliance during each VCO simulation. The normal and the stiff aorta's total *C_A_* and peripheral vascular resistance (*R_T_*) occurred by directly modifying the cardiopulmonary model systemic arterial parameters. Due to the large variance in vascular compliance and resistance between a human arterial system and a canine subject, the original canine normal and stiff Tygon arterial parameters were modified to align with the hemodynamics of the human cardiopulmonary model (Figure 3). The arterial compliances and resistances of the five compartments and aorta of the cardiopulmonary model were proportionally scaled to achieve a target equivalent compliance.

The normal canine total compliance was directly modified from a *C_A_* = 1.65 ml/mmHg to *C_A_* = 0.7 ml/mm Hg while the stiff Tygon compliance of *C_A_* = 0.19 ml/mm Hg produced physiologically accurate hypertensive conditions within the human cardiopulmonary model. Peripheral vascular resistance was modified from the canine normal *R_T_* = 3.04 mm Hg* ml^−1^ * sec^−1^ to *R_T_* = 1.28 mm Hg* ml^−1^ * sec^−1^ while the resistance of the stiff Tygon conduit was kept at the value of *R_T_* = 3.66 mm Hg* ml^−1^ * sec^−1^. The creation of the variables stroke volume, aortic flow and left ventricular pressures were the product of the Windkessel adaptation to the model.

Beat-to-beat data were collected by simulating the VCO under all levels of compliance. The results were verified through an examination of EDV, end-systolic volume (ESV), end-systolic pressure (Pes), end-diastolic pressure (EDP), PP, *dP*/*dt*_max_, PVA, and the ESPVR. The model results were validated by comparing the hemodynamic relationships seen in previous studies (14, 16, 18). Data analysis was conducted using GraphPad Prism (225 Franklin Street, Boston, MA, USA).

After calculating the normal and stiff data, the arterial compliance and resistance were linearly scaled starting from the normal compliance (*C_A_* = 0.7 ml/mm Hg and *R_T_* = 1.28 mm Hg* ml^−1^ * sec^−1^) and reduced to the Tygon compliance to verify the linear behavior of the cardiopulmonary model. The normal compliance *C_A_* was decreased by 10 percent to 0.63 ml/mm Hg and resistance of *R_T_* = 1.41 mm Hg* ml^−1^ * sec^−1^; then by 20 percent to *C_A_* = 0.56 ml/mm Hg and *R_T_* = 1.54 mm Hg* ml^−1^ * sec^−1^; then by 40 percent to *C_A_* = 0.42 ml/mm Hg and *R_T_* = 1.805 mm Hg* ml^−1^ * sec^−1^; and finally to the stiff Tygon compliance and resistance value of *C_A_* = 0.19 ml/mm Hg and *R_T_* = 3.66 mm Hg* ml^−1^ * sec^−1^. The model parameters and generated output are shown in Table 1.

**Table 1:** Impact of alterations in compliance on ventricular-vascular coupling.

Aortic Compliance (*C_A_*)	Normal	90%	80%	60%	Stiff
EDV (ml)	100.5	110.9	116.7	124.7	140.1
ESV (ml)	54.1	61.8	65.9	71.4	87.4
SV (ml)	46.5	49.1	50.8	53.3	52.7
SW (mmHg·ml)	3830	4802	5359	6150	7313
Pes (mmHg)	81.7	97.8	105.5	113.7	133.6
LVEDP (mmHg)	5.0	5.9	6.3	7.0	8.4
*dP/dt_max_* (mmHg/sec)	1621	1859	1967	2074	2057
PP (mmHg)	33.4	38.8	44.0	56.0	106.6
Art Ca (ml/mmHg)	0.57	0.50	0.48	0.47	0.39

EDV, left ventricular end-diastolic volume; ESV, left ventricular endsystolic volume; SV, stroke volume; SW, stroke-work; Pes, left ventricular endsystolic pressure; LVEDP, left ventricular end-diastolic pressure; *dP/dt_max_*, rate of rise of maximum left ventricular pressure over time; PP, pulse pressure; Art Ca, arterial compliance.

## 3 Evaluation of cardiac function

Like human and pre-clinical studies, left ventricular volume was calculated for each beat during the simulated VCO. The PVA, described initially by Suga et al. (22) and schematically represented in Figure 1, was calculated for each beat during the VCO. To calculate ESPVR, the relation was fit using a linear least-square algorithm to the equation:

$$P_{es}=\mathrm{ESPVR}/(V_{es}-V_{o})$$

where ESPVR is the slope of the relation and *V_o_* is its volume-axis intercept.

The slope and volume axis of the SW-EDV relation were determined using a linear least squares algorithm and were fit to the equation:

$$\mathrm{SW}=M_{W}/(\mathrm{EDV}-V_{W})$$

where *M_w_* is the slope of the relationship and *V_w_* is its volume axis (23).

The slope and volume axis of the *dP/dt*_max_-EDV relationship were determined in the same beats as used for the ESPVR and PRSW relations using a linear least squares algorithm and were fit to the equation:

$$dP/dt_{\max}=dE/dt_{\max}/(\mathrm{VED}\;\text{–}V_{o})$$

The slope of the *dP/dt*_max_-EDV relation, *dE/dt*_max_ represents the maximum rate of change of LV elastance (23). In a similar manner, the relation between PVA and EDV were fit to the equation:

$$\mathrm{PVA}=M_{\mathrm{PVA}}*(\mathrm{EDV}-V_{\mathrm{PVA}})$$

where *M*_PVA_ is the slope of the relationship and *V*_PVA_ its volume intercept.

The efficiency of left ventricular energy transfer (ME) was evaluated using the method of Nozawa et al. (24) using the equation:

ME=(SW/PVA)∗100

### 3.1 Evaluation of arterial compliance/elastance

**Figure 4:**
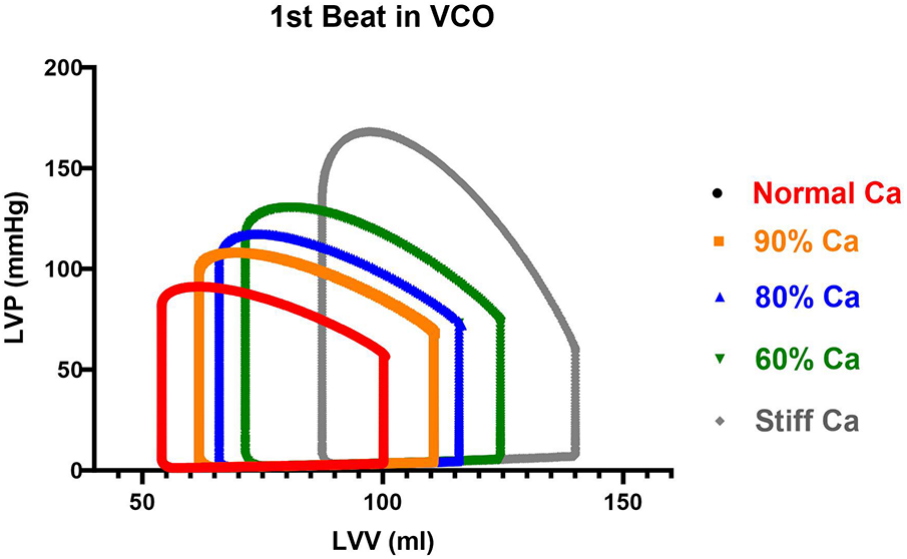
Impact of a Loss in Aortic Compliance on Steady State Cardiac Function. The first cardiac cycle from the VCO maneuver for each compliance is shown. LVV represents left ventricular volume, LVP represents left ventricular pressure.

**Figure 5:**
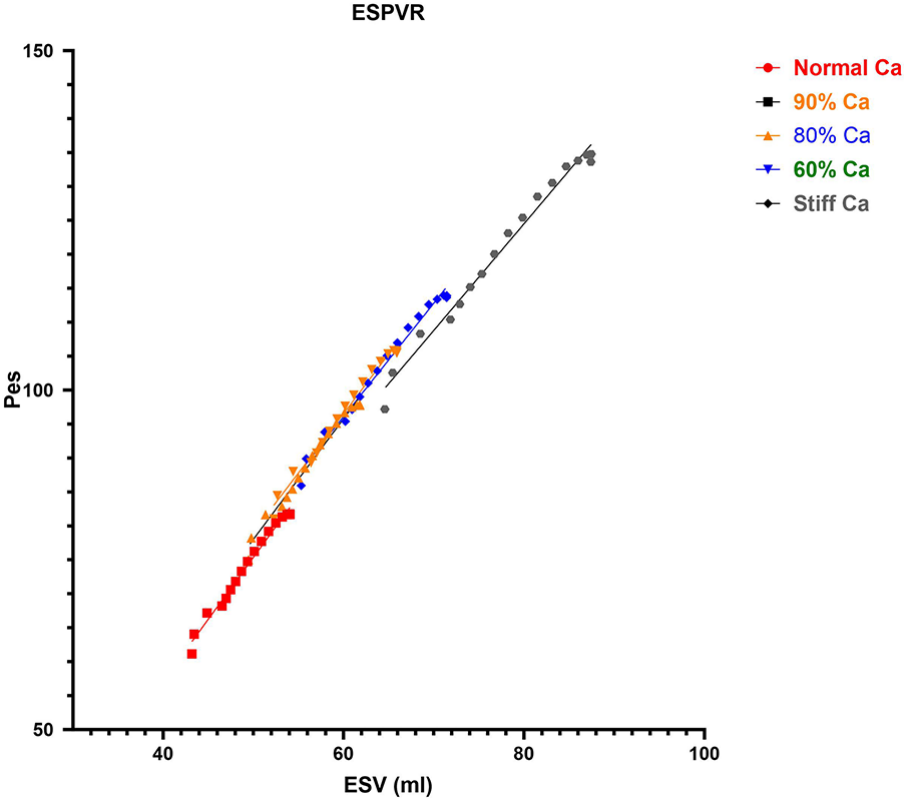
Impact of C_A_ on ESPVR. The data from the VCO maneuver for each compliance is shown. The slopes are similar but there was a small reduction in ESPVR at the Stiff C_A_. C_A_, aortic compliance; ESPVR, end-systolic pressure volume relationship; VCO, vena caval occlusion; Pes, end-systolic pressure (mmHg); ESV, end-systolic volume (ml).

The CM provided five levels of static arterial compliance as described above. In addition, calculations of dynamic arterial compliance (Art-cat: SV/Pes) was calculated. The parameters were calculated during the first beat of the VCO during steady-state (Figure 4) and transient state (Figure 5).

### 3.2 Evaluation of a loss in *C_A_*: phase I and II

Phase I data collection focused on beat-per-beat data obtained from simulating the VCO using the CM across the five levels of *C_A_* at 80 bpm. Phase II data collection focused on obtaining similar data at the normal and stiff *C_A_* at 60, 100, and 140 bpm, respectively. The results were verified through an examination of LV EDV, ESV, Pes, LVEDP, *dP/dt*_max_, PP, PVA, and ESPVR. The human CM results were validated by comparing the hemodynamic relationships seen in the work of Kelly et al. (14) and Moulton and Secomb (25) and physiological knowledge of the effects of stiffening vascular beds on the heart (14, 26).

## 4 Results

### 4.1 Phase I: impact of alterations in compliance on ventricular-vascular coupling

The changes in *C_A_* on LV function are shown in Table 2. The following hemodynamic changes occurred as *C_A_* decreased from normal to stiff. End-diastolic volume and the ESV gradually increased by 39.3% and 61.6% at the stiff setting. There was a minimal increase in SV from 46.5 to 52.7 ml. Changes in SW occurred in larger increments, from the normal to the stiff setting of 90.9%, and was mainly due to the significant increase in Pes. End-diastolic pressure increased from 5.0 to 8.4 mmHg during the normal to stiff setting, and end-systolic pressure increased from 81.7 to 133.6 mmHg. Pulse pressure increased from 33.4 to 106.6. The impact of the change in *C_A_* on the steady-state PV loop and cardiac function is pronounced, as shown in Figure 3.

**Table 2:** Impact of loss in aortic compliance on contractile state and ventricular-vascular coupling at 80 bpm.

Aortic Compliance	Normal (C_A_)	90%	80%	60%	Stiff
ESPVR	1.8	1.76	1.75	1.67	1.57
*dP/dt_max_*-EDV	16.9	17.2	17.1	16.7	14.1
PRSW	71.4	81.6	86.1	91.4	94.8
EDV-PVA	91.7	108.3	117.0	128.5	154.7
ME (%)	71.1	68.2	67.0	66.1	60.5

ESPVR, end-systolic pressure-volume relationship; *dP/dt_max_*-EDV, slope; PRSW, preload-recruitable stroke-work; EDV-PVA, end-diastolic volume pressure-volume area slope; ME, mechanical efficiency.

The loss in *C_A_* led to a loss in ME from 76.3 to 60.0% (Table 1). In addition, the EDV-PVA relation increased 59% from 91.7 to 154.7, while ESPVR demonstrate a small decrease 13% from 1.8 to 1.57 (Figure 5 and Table 1). The *dP/dt*_max_-EDV relation remained flat, and PRSW increased by 25% from 71.4 to 94.8, respectively. After the assessment of the physiological response of parameters describing cardiac function across the range of *C_A_* and a response to pre-clinical data and the recent work by Moulton and Secomb (25), the Phase II component was designed to build on Phase I.

### 4.2 Phase II: impact of alterations in compliance and heart rate on ventricular-vascular coupling

Phase II evaluated how a change in heart rate (the force-frequency effect at the normal and stiff *C_A_* impacted the measures of cardiac function and ME as described above. The impact of increases in HR from 60, 100, and 140 bpm at the normal and stiff *C_A_* is shown in Table 3. From the baseline HR of 60 bpm, EDV decreased 47% from 113.2 to 75.4 ml at 100 bpm in the normal setting and decreased 52.7% from 159.6 to 75.4 ml in the stiff setting at 140 bpm. Similarly, ESV decreased 11.2% from 54.7 to 48.6 ml at 100 bpm and 47.7% from 92.9 to 48.6 ml at 140 bpm. This change resulted in a large decrease in SV from 58.5 to 26.7 ml (54.3%) and 59.9% from 66.6 to 26.7 ml, at 100 and 140 bpm, respectively. In the normal *C_A_* setting, as HR increased, a small decrease occurred in end-systolic pressure from 82.8 to 72.1, while LVEDP remained stable. The change in end-systolic pressure was much greater in the stiff *C_A_* setting compared to the normal setting, decreasing from 141.1 to 119.5 mmHg from 60 to 140 bpm, with a nearly two-fold increase in LVEDP from 5.5 to 9.9. The loss in SW in both the normal and stiff settings, as HR increased, was similar between the two settings at 62%.

**Table 3:** Impact of increases in heart rate and a decrease in aortic compliance on steady state cardiac function.

Aortic compliance	Normal			Stiff		
HR	60	100	140	60	100	140
EDV (ml)	113.2	86.9	75.4	159.6	123.2	75.4
ESV (ml)	54.7	49.6	48.6	92.9	78.3	48.6
SV (ml)	58.5	37.3	26.7	66.6	44.8	26.7
Pes (mmHg)	82.8	73.3	72.1	141.1	119.4	72.1
LVEDP (mmHg)	5.5	5.6	6.3	9.9	8.3	9.4
PP (mmHg)	31.1	25.4	22.4	77.5	62.4	56.6
Art Ca	0.71	0.51	0.37	0.47	0.38	0.28
*dP/dt*_max_ (mmHg/sec)	1,086	1,003	1,050	1,357	1,260	1,403
SW (mmHg·ml)	5,078	2,788	1,923	10,322	5,684	3,989

HR, heart rate; EDV, left ventricular end-diastolic volume; ESV, left ventricular end-systolic volume; SV; stroke volume; SW, stroke-work; Pes, left ventricular end-systolic pressure; LVEDP, left ventricular end-diastolic pressure; *dP/dt*_max,_ rate of rise of maximum left ventricular pressure over time; PP, pulse pressure; Art Ca, arterial compliance.

The force-frequency relationship was intact in both settings. The change in the EDV-PVA relation from 60 to 140 bpm was greater during the stiff setting (182.1 vs. 141.0 vs. 131.2) compared with the normal setting (100.6 vs. 83.2 vs. 79.1) (Table 4). The change in *dP/dt*_max_-EDV relation from 60 to 140 bpm was slightly lower during the stiff setting (6.5 vs. 9.3 vs. 12.9) compared with the normal setting (7.25 to 10.1 to 14.0) across the HRs. The change in ESPVR from 60 to 140 bpm increased more in the stiff setting (1.45 vs. 1.75 vs. 1.92) compared the normal setting (1.75 vs. 1.99 vs. 2.0). Finally, the change from the normal to the stiff setting impacted PRSW to the greatest degree for all four parameters. During the stiff setting, the impact of HR was greater (118.5 vs. 72.7 vs. 54.6) compared to the normal setting (82.5 vs. 63.8 vs. 56.5).

**Table 4:** Impact of increases in heart rate and a decrease in aortic compliance on contractile state.

Aortic compliance	Normal			Stiff		
HR	60	100	140	60	100	140
ESPVR	1.75	1.99	2.0	1.45	1.75	1.92
*dP/dt*_max_-EDV	7.3	10.1	14.0	6.5	9.3	12.9
PRSW	82.5	63.8	56.5	118.5	72.7	54.6
EDV-PVA	100.6	83.2	79.1	182.1	141.0	131.2
ME (%)	76.4	69.9	62.6	65.8	60.7	53.2

HR, heart rate; ESPVR, end-systolic pressure-volume relationship; *dP/dt*_max_-EDV, slope; PRSW, preload-recruitable stroke-work; EDV-PVA, end-diastolic volume pressure-volume area slope; ME, mechanical efficiency.

### 4.3 Phase I and II: impact of alterations in compliance on dynamic compliance

The impact of the changes in *C_A_* on VVC is shown in Tables 1, 2, with dynamic compliance [i.e., arterial compliance (Art-ca)] and the EDV-PVA relation providing insight. During the decrease in *C_A_*, in Phase I, Art-ca decreased 31.5% from 0.57 to 0.39, respectively. In Phase II, a loss in Art-ca across the values of *C_A_* led to a large increase in the EDV-PVA relation of 68.7% from 91.7 to 154.7. The impact of increases in HR on the changes in Art-ca across the levels of *C_A_* was also large. From 60 to 140 bpm, Art-ca decreased from 0.71 to 0.37, a loss of 47.8% at the normal *C_A_*. At the stiff *C_A_*, from 60 to 140 bpm, the Art-ca began at a lower setting, 0.47, and decreased further at the stiff setting to 0.28, a loss of 40.4%. The EDV-PVA for the normal *C_A_* at 60 bpm was 100.8 and decreased 21% to 79.1%, while the loss was greater at the stiff *C_A_* (182.1 to 131.2).

## 5 Discussion

### 5.1 Model rationale

The *in-silico* CM was modified to evaluate three questions: (1) Does the model simulate VA across the compliance spectrum for the human circulatory system in the pressure-volume construct; (2) Does the model generate LV pressure-volume data that agrees with previous studies; and (3) Does the model mimic the in-vivo response to elevated heart rates. To accomplish this, we modified a lumped parameter approach. The original model did not include components to quantify or modify biochemical features of cardiac function that change with aging, arterial wave reflections, local vessel impedance, or changes in microvascular function that occur with aging and co-morbid conditions. The current model provides insights into how VA impacts cardiac function and energetics. The three objectives noted above were achieved, and the model generated PV loop data congruent with previous studies and added to our understanding of the impact of VA on cardiac function and energetics.

The data suggest the following: first, the model successfully produced a physiologic response in parameters commonly used to describe ventricular function and VVC. Second, the responses were graded as the model compliance in the model was reduced and provided insight into how the aging aorta may impact cardiac function. The impact of simulated VA on ME was shown to agree with previous work (14, 16, 18). Third, the results regarding the CM's ability to simulate VA agree with the results of the decades-long Framingham Study regarding how a loss in *C_A_* may lead to a gradual increase in PP in a healthy portion of the population (26). Fourth, the model provided the initial view of how a loss of *C_A_* coupled with an increase in HR impacts ME. Finally, the CM agrees with recent cardiac modeling work by Moulton and Secomb (25).

Over the past fifty years, complex studies using various methods have concluded that changes in compliance impact ventricular function (8, 9, 15). The recent observations from the Framingham Study provide key epidemiological evidence supporting the previous *in-vivo* studies that a decline in compliance is related to changes in left ventricular function (26). The Framingham studies have also shown that a decrease in compliance results in an increase in PWV, which has a negative impact on microvascular beds (12). The current model did not calculate PWV and does not include tools to quantify microvascular changes that would accompany the change in model compliance.

### 5.2 Ventricular-vascular coupling

 The *in-vivo* studies by Kelly et al. (14) and Freeman have shown that decreasing compliance causes a large increase in PP and end-systolic pressure without impacting ESPVR, leads to increased left ventricular EDV and ESV, and results in a loss in ME (18). Conversely, increases in compliance results in lower PP, SW, PVA without a change in ESPVR or EDV-PVA (16). A closer review of the data collected by Kelly et al. (14) demonstrated that a large decrease in compliance (8-fold) resulted in no change in ESPVR or PRSW. With the individual animal data shown in Table 1 of the publication, the range of responses from normal to stiff compliance shows that the variability within the *in-vivo* setting produces findings that require more insight. Six of the nine dogs showed an increase in ESPVR from 5.8 to 8.0 mmHg/ml, a 34% increase with a loss in *C_A_* and in the remaining three dogs, ESPVR decreased from 8.7 to 5.4 mmHg/ml, a 33% decrease, with a similar change in the chronically instrumented animal model by Freeman.

A similar observation was seen with PRSW but not with the same dogs responding as they did regarding ESPVR, an expected response in this complicated animal model. Overall, the change or lack of change in ESPVR or PRSW occurred with an overall increase in MVO_2_ with the Tygon aorta. This *in-vivo* model suggests that the loss in compliance resulted in an energetic and mechanical loss without a change in contractile state. Comparing this data to the data collected by Freeman, the canines were evaluated at their normal compliance and decreased compliance following the inflation of a 10 ml Fogarty catheter in the proximal descending aorta. Left ventricular EDV did not change with the balloon inflated for 5 min, while *dP/dt*_max_ increased significantly along with an increase in PVA and a loss in ME (18).

In a similar manner to that observed in the work by Kelly et al. (14), ESPVR and PRSW were unchanged during inflation of Fogarty balloon (18). The lack of change in ESPVR and PRSW occurred while the EDV-PVA variable was significantly higher with the balloon inflated (107.5 ± 17.3 vs. 196.0 ± 29.5 erg·10^−3^·cm^3^) (18). This contrasts with the data by Kohl et al. (16) where an increase in compliance did not impact the EDV-PVA construct. The differences in these observations may be due to the animal model in addition to the method used to alter compliance. The current CM data agrees with several findings from each of these studies. The CM builds on the previous work and demonstrates that in the presence of a healthy heart, simulated VA impacts the variables in a physiologically expected manner.

Changes in cardiac function due to the presence of noted risk factors may occur or be subclinical for several decades (27). This may occur without measurable changes in blood pressure or other measures of cardiovascular disease. In patients with early vascular aging, it is likely that loss in compliance will create measurable change in cardiac function and microvascular function (28). As compliance decreases, the work of the heart increases, the sub-endocardium becomes vulnerable to ischemia, and the renal and cerebral vascular beds are subjected to pulsatility, leading to the development of dysfunction in these organs (29, 31).

## 6 Limitations

Previous models have used a combination of complex ventricular and vascular components employing both finite element and lumped parameter approaches. The current model, based on the original work, permits evaluation of cardiac function during simulated vascular aging based on an integrated three-element Windkessel. Our model did not include coding or parameters to alter the impact of wave reflection on VVC. While pulse-wave velocity is an important parameter for use in the clinical setting, its addition to the current model will likely correlate well with changes in *C_A_*. The ventricular component was based on the time-vary elastance construct for the normal heart (31). Opportunities to evaluate states of left ventricular dysfunction, the impact of wave reflections and coronary dysfunction are possible improvements to the model.

## 7 Conclusion

This *in-silico* model provided novel insight into how VA impacts the PV loop construct parameters. The findings in this study established observations regarding the impact of VA on PV-derived variables, including the contractile state, SW, and ME. The model's speed and data and analysis generation were similar to conducting *in-vivo* studies.

## Data Availability

The original contributions presented in the study are included in the article. Further inquiries can be directed to the corresponding author.
